# Functional anatomy of the precibarial valve in *Philaenus spumarius* (L.)

**DOI:** 10.1371/journal.pone.0213318

**Published:** 2019-02-28

**Authors:** Sara Ruschioni, Emanuele Ranieri, Paola Riolo, Roberto Romani, Rodrigo P. P. Almeida, Nunzio Isidoro

**Affiliations:** 1 Department of Agricultural, Food and Environmental Sciences, Marche Polytechnic University, Ancona, Italy; 2 Department of Agricultural, Food and Environmental Sciences, University of Perugia, Perugia, Italy; 3 Department of Environmental Science, Policy and Management, University of California, Berkeley, CA, United States of America; University of Catania, ITALY

## Abstract

In phytophagous sap-sucking insects, the precibarial valve plays an important role in sap ingestion. We used light and electron microspcopy to study the morphology and the ultrastructure of the precibarial valve of the meadow spittlebug, *Philaenus spumarius* (Hemiptera, Aphrophoridae), in order to better understand the operative mechanism of this structure. The precibarial valve revealed to be a complex structure with a bell-like invagination in the middle of the precibarium (on the epipharynx). Unlike the current hypothesis, we propose that the valve opens by dilator muscles and closes through cuticular and fluid tensions, the latter leading to morphological changes to the plane of the valve based on sap flow. Moreover, the presence of a precibarial secretory structure is described for the first time for auchenorrhynchan insects. In light of these observations, functions are hypothesized and discussed for this secretory structure.

## Introduction

In phytophagous sap-sucking insects, mouthpart adaptations allow for feeding on hosts by inserting stylets into specific tissues (e.g. phloem, xylem) [1–4]. The ingested sap passes through the suction duct (obtained by the two coapted maxillary stylets) to the cibarium, then reaching the esophagus [5]. The cibarium is a pump chamber with rigid cuticle in the ventral side and a flexible membranous diaphragm, to which membrane dilatator muscles are dorsally attached. Active sap ingestion is made possible thanks to this diaphragm pump [5,6]. When these muscles contract the diaphragm elevates, the pump volume increases, and the pressure lowers within the chamber relative to the sap on which the insect is feeding [6–9]. The region of the alimentary canal between the divergence point of the stylets and the cibarium is termed precibarium. This canal is formed by the apposition of the epi- and hypopharynges [6,10–13]. Sap flow is controlled by two valves: the anterior precibarial valve and the esophageal valve. When the diaphragm is lifted up, the precibarial valve opens and fluids are sucked up into the cibarium. When the cibarial muscles relax, the diaphragm collapses to the bottom of the cibarium, pushing fluids into the esophagus [8,9]. At that time, the precibarial valve is closed and the the esophageal valve, that is located between the esophagus and the mesenteron, passively opens due to positive pressure, as fluid accumulated in the cibarium is compressed during cibarial muscle relaxation [9,10].

The precibarial valve plays an important role in sap ingestion. It can stop fluid from being drawn immediately up the food canal when the insect probes; it maintains the pressure differences required for continuity of the fluid movement in the foregut. The precibarial valve also functions in regulating or reducing fluid uptake directly, in particular in regulating the flow of hydrostatic fluids [6,10,11,14]. The presence of the precibarial valve in Hemiptera was described first in aphids by Ponsen [15], afterwhich Backus and McLean [6] hypotesized that the valve may be present in all Sternorrhyncha and Auchenorrhyncha. On the basis of morphological investigations, it is generally accepted that the basic mechanism of sap ingestion is similar in leafhoppers [6,10], aphids [14], psyllids [14] and thrips [7].

The meadow spittlebug, *Philaenus spumarius* L. (Hemiptera: Aphrophoridae), is a ubiquitous species with a wide Holarctic distribution [16]. Nymphs and adults are xylem-sap feeders [17,18]. The meadow spittlebug feeds on hundreds of host plants [19,20]. In Europe, *P*. *spumarius* is not considered a pest [21,22], but recently this species was reported as vector of *Xylella fastidiosa* associated with olive quick decline syndrome in the Salento Peninsula (Southern, Italy) [23–25]. Considering the recent applied interest in *P*. *spumarius* as a pathogen vector, as well as the potential role of sap ingestion dynamics by these insects on transmission of *X*. *fastidiosa*, we revisited the morphology of the foregut of this insect, focusing on the precibarial canal. Our results suggest that the functional mechanism of the precibarial valve of *P*. *spumarius* might differ from the previously proposed models. We also report the presence of a novel secretory structure associated with that region of the foregut of *P*. *spumarius*.

## Materials and methods

### Insects

Adults of *P*. *spumarius* were collected from known plant hosts [20,26,27] in olive orchards of the Ancona district (central-eastern Italy), from May to June 2017, using a modified leafblower (Tanaka Togyo Co., THB-2510) in which the intake port was fitted with a fine mesh organza bag. Captured specimens were positioned in a cage (Bugdorm-I, Megaview) with wet paper and fresh host plant (lemon balm and clover) shoots, until arrival at the laboratory.

### Light microscopy

*P*. *spumarius* specimens were prepared following TEM preparation in Ruschioni et al. [28]. Adult insects were anesthetized by exposure to cold temperatures (-18°C) for 60 seconds, then immediately immersed into a solution of glutaraldehyde 2% and paraformaldehyde 2.5% in 0.1 M cacodylate buffer +5% sucrose, pH 7.2–7.3. Each head capsule was detached from the rest of the body and sectioned with a scalpel blade along the fronto-clypeus sutures. Each specimen was immersed in the fixative for 2 hours at 4°C, and transferred to 0.1 M cacodylate buffer +5% sucrose, pH 7.2–7.3 overnight at 4°C. Heads were post-fixed in 1% OsO_4_ (osmium tetroxide) for 1 hour at 4°C and rinsed in the same buffer. Dehydration in a graded ethanol series from 60% to 99% (15 minutes each) was followed by embedding samples in Epon-Araldite with propylene oxide as bridging solvent. Semi-thin sections were obtained with a diamond knife on a LEICA ULTRACUT R ultramicrotome (Leica), mounted on slides, and stained with Toluidine blue for 1 min at room temperature. Slides were observed using a model Nikon Eclipse E600 microscope (n = 20; sex ratio, 1:1).

### Scanning electron microscopy

*P*. *spumarius* specimens were prepared following Ruschioni et al. [28]. Adult were anaesthetized by exposure to cold temperature (-18°C) for 60 seconds, and then dipped in 50% alcohol. Individuals were dissected cutting at the level of the neck, removing the head capsule from the body, and heads dehydrated in an ethanol series (60% to 99%) for 15 min each. After dehydration, 99% ethanol was substituted with pure HMDS (Hexamethyldisilazane, Sigma) and the specimens were allowed to dry in a hood at room conditions. On each aluminum stub, 5 samples were mounted placing the precibarium upwards in such a way as to make it clearly visible to the SEM observations. Observations were carried out using a FE-SEM Zeiss SUPRA 40 (Carl Zeiss NTS GmbH, Oberkochen, Germany). (n = 30; sex ratio, 1:1)

### Transmission electron microscopy

*P*. *spumarius* specimens were prepared following Ruschioni et al. [28]. Adults insects were prepared following the same method used for light microscopy. Thereafter, the samples were sectioned with a diamond knife on a LEICA ULTRACUT R ultramicrotome (Leica), and mounted on formvar coated 50 mesh grids. Sections on grids were stained with uranyl acetate (20 min, room temperature) and lead citrate (5 min, room temperature). Finally, the sections were analyzed with a Philips EM 208. Digital pictures (1376 x 1032 pixels, 8b, uncompressed greyscale TIFF files) were obtained using a high-resolution digital camera MegaViewIII (SIS) connected to the transmission electron microscope.

## Results

### Morphology of the precibarial valve

**Fig 1:**
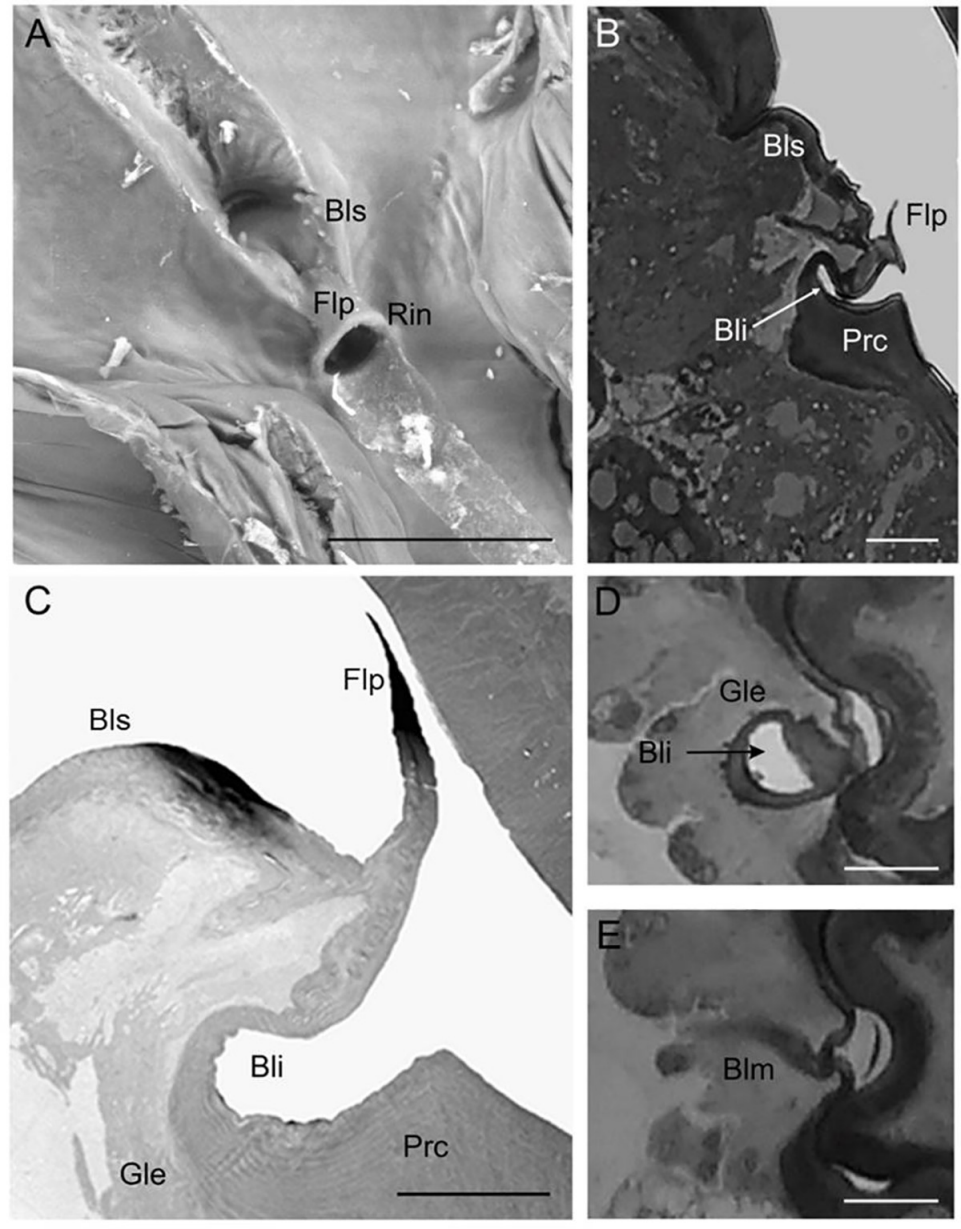
Precibarial valve of *Philaenus spumarius*. (A) SEM image of the epipharynx at the level of the precibarial valve showing the rigid ring (Rin) from which the bell-like invagination folds up in the head cavity, the cuticular flap (Flp) and the basin-like structure (Bls). (B) LM longitudinal section of the epipharynx showing the bell-like invagination (Bli), the cuticular flap (Flp), the basin-like structure (Bls) and the procuticle (Prc). (C) TEM longitudinal section at the level of the bell-like invagination in which is visible the thick procuticle (Prc) and a small portion of the gland (Gle). (D) LM cross-section taken below the level of the ring, the cuticle forming the bell-like invagination (Bli) and the glandular epithelium (Gle) are visible. (E) LM cross section at the level of the basin-like structure in which is visible part of the muscle (Blm). Scale bar: a = 25 μm; b,d,e = 20 μm; c = 10 μm.

In *P*. *spumarius*, the precibarium is formed by the juxtapositon of the hypopharynx and the epipharynx. The medial region of the precibarium showed the presence of a valve (Fig 1A). We observed that the precibarial valve is a bell-like invagination placed in the middle of the epipharynx (Fig 1A–1C). Moving from the stylets to the cibarium, the epipharyngeal lumen formed a rigid ring from which a bell-like invagination fold up in the head cavity towards the clypeus, slightly bent in the direction of the cibarium (Fig 1A–1C). Distally, the ring hosted a rigid flat cuticular flap, that extended towards the cibarium (Fig 1A–1C). This flap showed a curved shape that morphologically mirrors the hypopharyngeal concavity (Fig 1A, 1B, 1D and 1E). Behind the flap, moving toward the cibarium, a basin-like structure was formed (Fig 1A–1C), after which a smooth surface made by a very thick cuticle continued with the cibarium (Fig 1A).

The cuticle of the epipharynx varied in thickness (Fig 1B and 1C). While the epicuticle (outermost layer of the cuticle) was similar in thickness (Fig 1B and 1C), the procuticle (inner part made up by ten layers; Fig 1C), was highly variable in thickness (Fig 1B and 1C). Finally, LM and TEM showed the presence of a muscle (Fig 1B,1C and 1E) inserted at the level of the basin-like cuticle and forming a bundle connected with the clipeum.

### Secretory gland associated with precibarial valve

**Fig 2:**
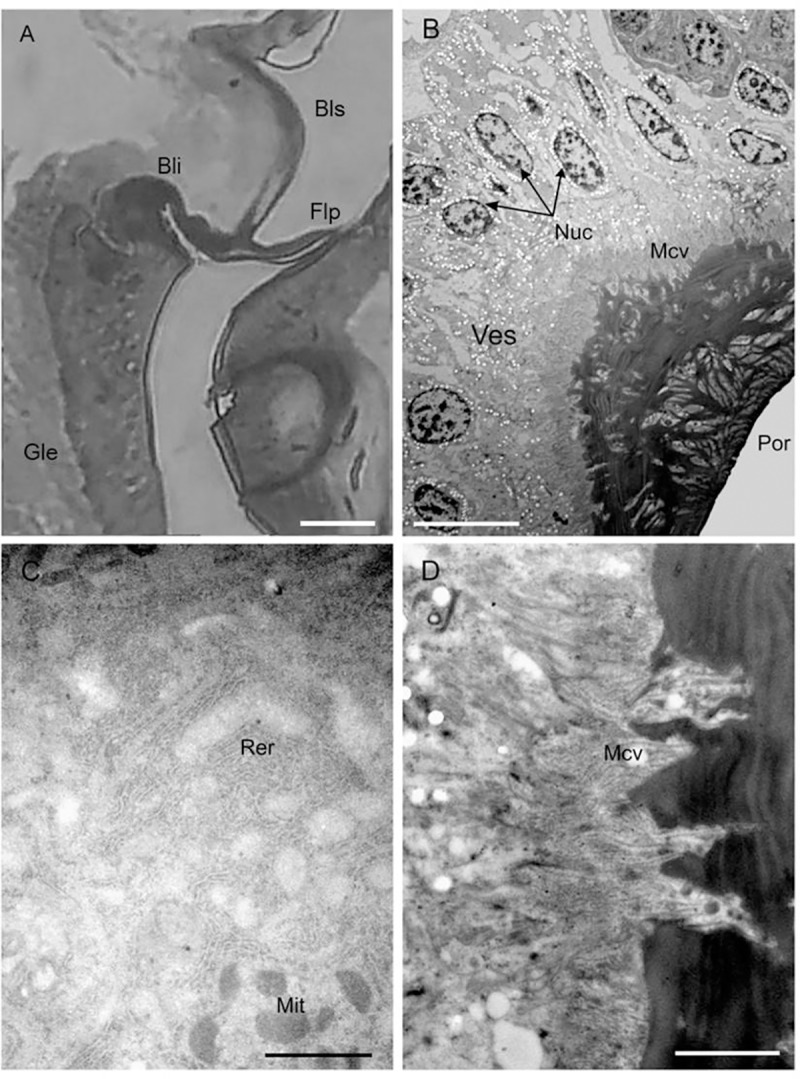
Precibarial gland of *Philaenus spumarius*. (A) LM longitudinal section of the epipharynx taken at the level of the bell-like invagination (Bli) showing the large epithelial structure (Gle); (B) TEM detail of glandular epithelium with the the secretory cells, showing abundant apical microvilli (Mc.v), nuclei (Nuc), small electron-lucid vesicles (Ves) and the release area with the canal pores (Por); (C) TEM close-up view of the cytoplasm of a secretory cell, showing rough endoplasmic reticulum (Rer) and mitochondria (Mit); (D) TEM detail of the apical region of the secretory cells, showing abundant microvilli arising from the cuticle. Bls: basin-like structure; Flp: flap. Scale bar: a = 25 μm; b = 5 μm; c = 1 μm; d = 2 μm.

A large epithelial structure was observed within the lumen of the epipharynx, starting from the bell-like invagination and extending distally (Figs 1D and 2A). The cells were in close contact with the internal cuticular wall (Figs 1D, 2A and 2B). The epithelium was made up of a single layer of large secretory cells (Fig 2B). The cytoplasm of the cells had numerous small electron-lucid vesicles (Fig 2B and 2D), as well as rough endoplasmic reticulum (Fig 2C) and mitochondria (Fig 2C). The apical region of the cells had microvilli connected with the innermost cuticular layer (Fig 2B and 2D). The cuticle in the inner part was more compact, while the outer region showed a sponge-like structure, perforated with small pores (Fig 2B).

## Discussion

The precibarial valve is a key structure regulating plant sap ingestion by hemipteran insects [6,10,11,14]. In aphids the precibarial valve was described as bulbous with a deep central suture, attached to a piston and a group of dilator muscles attached to anteclypeus [11]. In psyllids the valve is flap-like, but folded and unhinged, similarly attached to a piston and dilator muscles [14]. In leafhoppers the same valve was described as a flap-like and hinged structure, attached to a group of muscles originating on the clypellus. In the leafhopper *Macrosteles fascifrons* (Hemiptera: Cicadellidae) it was proposed that the precibarial valve has a bulbous tip that is attached to the proximal side of a pit; when open, the valve fits into a groove at the anterior end of the basin [6]. Differently, our observations with *P*. *spumarius* indicate that the precibarial valve is a complex structure, with a bell-like invagination located in the middle of the epipharynx, linked to a flap-like structure that pivot on it, attached to a muscle originating on the clypellus. In other words, our interpretation differs from prior studies with related insects (Cicadellidae), where the flap was described as the precibarial valve itself [6–10]. We note that the morphology of the precibarial region of leafhoppers [6,10] is similar to that of *P*. *spumarius*, therefore our interpretation of the morphology and functioning of the precibarial valve could be shared with leafhoppers as well.The operative mechanism of the precibarial valve was described as a muscle dependent structure [11,14], with muscles forming a bundle originating on the inner face of the clypellus and entirely separated from the cibarial muscles, indicating the likelihood of its independent action [6,10,11,14]. However, there exist an important discrepancy in regards to the functioning of the precibarial valve in relation to its muscles. In other words, the mechanism of opening and closing of the valve has been differentially described for some insect groups. In aphids [11], psyllids [14] and, whiteflies [8], the precibarial valve opens when the precibarial muscle contracts, while it closes against the valve’s receptable when the muscle relaxes. In leafhoppers, the opposite has been proposed. When the precibarial muscle contracts to pull the apodeme, the valve is pushed outward to fill the lumen closing off the canal, while opening of the precibarial canal is accomplished by relaxation of the muscle and associated movement of the precibarial valve away from the hypopharyngeal surface [6,10]. Our observations suggest that the functioning of the precibarial valve in leafhoppers could be in fact similar to that of sternorrhynchan insects.

**Fig 3:**
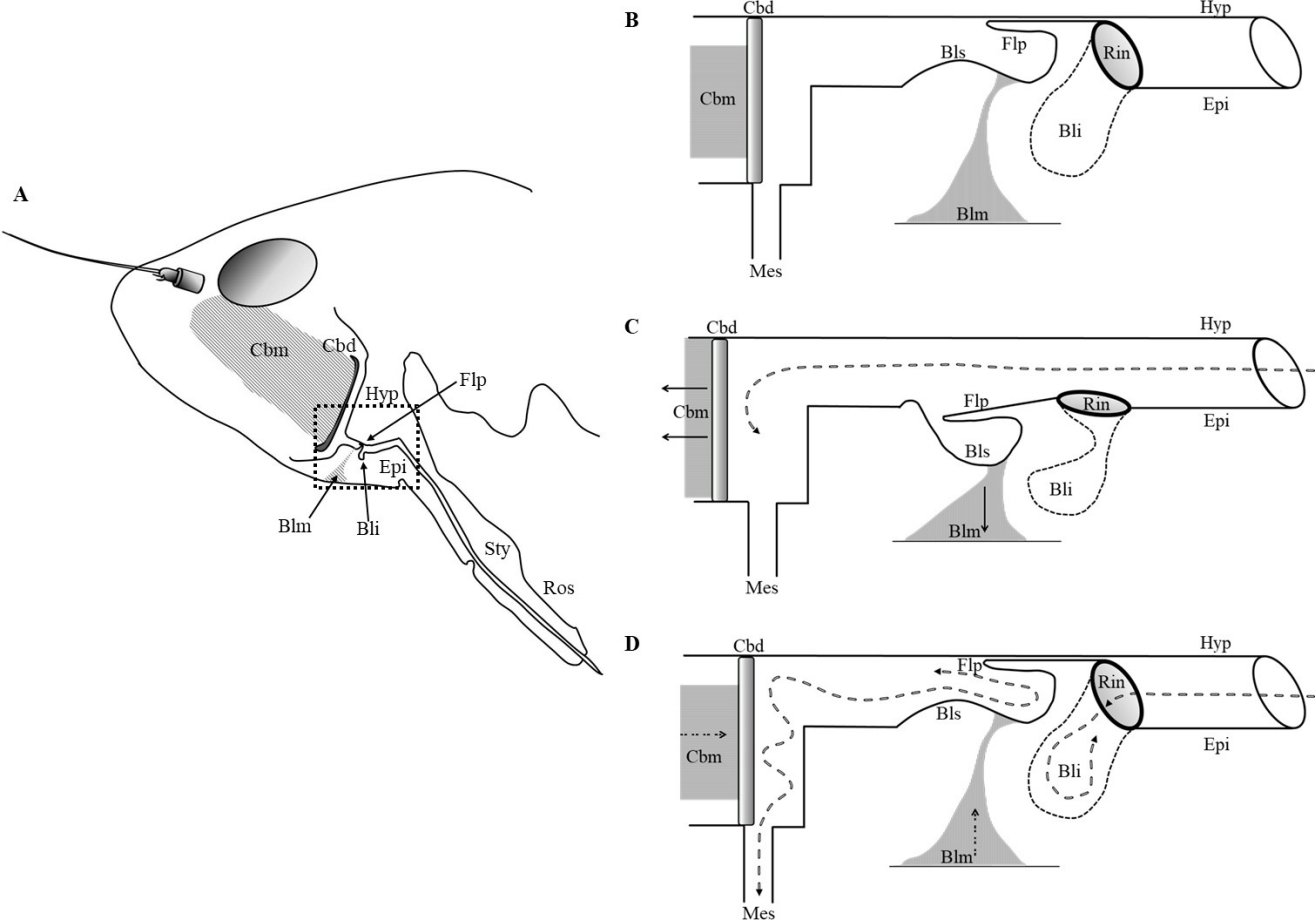
Operative mechanism of the precibarial valve in *Phylaenus spumarius*. (A) Schematic drawing of the mouthpart of *Philaenus spumarius* showing the rostrum (Ros), the stylets canal (Sty), the hypopharynx (Hyp) and the epipharynx (Epi), the cibarium with its muscle (Cbm) and diaphragm (Cbd) and the precibarial valve in which is depicted the bell-like invagination (Bli), the flap (Flp) and the basin-like muscle (Blm). The dashed square represents precibarial area below schematized at three different steps during fluid uptake: at the relaxing state with the valve closed (first step); during the feeding, with the valve opened (second step); when the cibarium chamber is filled up and the valve is closed (third step). (B) First step: relaxing state, the valve is close. The flap (Flp) lays aligned to the hypopharynx (Hip), the muscles (Cbm, Blm) are relaxed; (C) Second step: the insect starts feeding and the valve opens. The cibarial muscle (Cbm) contracts (solid lines), pulling the food pump open. The basin-like muscle (Blm) contracts (solid lines), bringing along the flexible cuticle of the basin-like structure (Bls), then the ring (Rin) bends towards the cibarium, pivoting on the base attached to the distal surface of the lumen and the flap (Flp) is lowered and leaned to the basin-like structure (Bls). At that time, the sap is free to pass by (dashed line); (D) Third step: the cibarium chamber is filled up, the valve closes. Shortly before the downward strokes of the cibarial diaphragm (Cbd), the basin-like muscle (Blm) relaxes (dotted lines), then the flexible cuticle of the basin-like structure (Bls) return in its original position. Hence, also the flap (Flp) moves up and goes towards the hypopharynx (Hyp) and the ring (Rin) tends to turn back to its original position and starts to occupy the lumen of the precibarium. The sap starts to flow inside the bell-like invagination (Bli), filling it up and creating a pressure that push the flap (Flp) against the hypopharynx (Hyp). When the precibarial valve is close, the cibarium pumps the sap towards the mesenteron (Mes), and part of it is pushed towards the precibarial valve. The pressure of the fluid (dashed line) pushes it into the internal wall of flap (Flp), forcing it to be attached to the hypopharynx (Hyp).

Based on the morphological features of *P*. *spumarius* described in this work we hypothesize the operative mechanism of its precibarial valve (Fig 3). In a relaxed state, the precibarial valve is closed (Fig 3B); the flap sits against the hypopharynx thanks to its curved shape that mirrors the hypopharyngeal concavity, the ring is lift, the basin-like structure is relaxed and rounded. When the insect initiates sap ingestion (Fig 3C) the cibarial muscles contract pulling the cibarial pump open (as described in Backus and McLean [6]). Concurrently the basin-like muscle of the precibarial valve contracts, retracting the flexible cuticle of the basin-like structure. Presumably, the thickness of the procuticle here decreases in order to confer more elasticity. As a consequence the whole structure bends. The ring bends towards the cibarium, pivoting on the base attached to the epipharynx, then the flap is lowered and leans against the basin-like structure, and the bell-like invagination compresses. As a result, the valve ‘opens’ and plant sap pass through the precibarial canal. When the cibarium chamber is filled (Fig 3D), the cibarial diaphragm moves downward due to the cibarial muscle relaxation, pumping the sap towards the esophageal valve [6]. At that moment, the precibarial valve necessarily needs to be closed in order to avoid sap from being expelled via the stylets. The bell-like invagination has the function of facilitating the closure of the valve. Shortly before the downward strokes of the cibarial diaphragm, the basin-like muscle relaxes, and the flexible cuticle returns in its original position and the flap moves back towards the hypopharynx. The relaxation of the muscle alone cannot guarantee the complete closure of the valve, an antagonistic muscle would be necessary, as the force of sap flow passing through the precibarium channel would likely prevent complete adhesion of the flap against the hypopharynx. We hypothesize that the morphology of the bell-like invagination helps in closing the valve. The relaxation of the precibarial valve muscle enables the ring to return to its original position. When the ring starts to occupy the lumen of the precibarium, sap gradually flows into the bell-like invagination, generating enough pressure to push the flap against the hypopharynx, leading to complete closing of the valve and the precibarial canal. The stiffness of the ring is important in avoiding collapse of the bell-like invagination, otherwise the structure would not function. Thereafter, the cibarium pumps the sap towards the mesenteron [6]. At the same time, part of the liquid is pushed towards the precibarial valve, hence the under-pressure fluid is pushed towards the internal wall of the flap, causing the whole structure to be forced towards the hypopharynx. At that point, the valve is completely closed (Fig 3D).

We also report the presence of a large epithelial structure within the lumen of the epipharynx, which starts from the bell-like invagination and extends distally. According to the classification of insect epidermal glands proposed by Quennedey [29], the secretory cells associated with the precibarium in *P*. *spumarius* belong to class 1, as they are in direct contact with the external cuticle and lack specialized transporting cells or cuticular ducts. The cytoplasm of the cells showed numerous small electron-lucid vesicles, as well as rough endoplasmic reticulum and mitochondria, typical of cells characterized by an intense secretory activity. Although further studies are needed to understand the chemical nature of the substances secreted by the glands and the possible functions of the gland, we propose a series of hypothesis. First, secretions could assist in keeping the bell filled and consequently the precibarial valve closed. Alternatively, the secretions may contain an array of proteins, including effectors or enzymes, which may assist insects in sap ingestion. Because *P*. *spumarius* feeds on a dilute diet that is under negative tension, the force exerted by the cibarial pump is high. The secretion of the gland could have a lubrificant function and decrease the sap friction between the canal wall and the liquid, making the liquid flow more energetically efficently.

In conclusion, we described the morphology and the possible operative mechanism of the precibarial valve in *P*. *spumarius*. The ultrastructural analysis carried out on the precibarium helped us characterise this complex structure, and to propose a hypothesis on the functioning of the precibarial valve during sap ingestion by auchenorrhynchan insects.
